# Mapping the Process of Engagement With Digital Health Interventions: A Cross-Case Synthesis

**DOI:** 10.1016/j.mayocpiqo.2025.100625

**Published:** 2025-05-27

**Authors:** Madison Milne-Ives, Sophie R. Homer, Jackie Andrade, Edward Meinert

**Affiliations:** a Faculty of Medical Sciences, Translational and Clinical Research Institute, Newcastle University, United Kingdom; b Faculty of Health, Centre for Health Technology, University of Plymouth, United Kingdom; c Faculty of Health, School of Psychology, University of Plymouth, United Kingdom; d Department of Primary Care and Public Health, School of Public Health, Imperial College London, United Kingdom

## Abstract

**Objective:**

To map the associations between affective, cognitive, and behavioral components of engagement with digital health interventions to provide a framework to improve intervention design, evaluation, and impact.

**Patients and Methods:**

An exploratory multiple case study examined 3 studies evaluating a childhood obesity mobile application (NoObesity, data collection: from September 15, 2020 to June 23, 2021), a mental health conversational agent mobile application (Wysa, data collection: from December 13, 2022 to July 31, 2023), and a telephone-delivered conversational agent postsurgical assessment (Dora R1, data collection: from September 17, 2021 to January 31, 2022). Qualitative data from semi-structured interviews (NoObesity: n=15, Wysa: n=4, and Dora R1: n=20) was analyzed using a codebook thematic analysis approach to generate models mapping engagement. A cross-case analysis compared the 3 models with a hypothesized model.

**Results:**

The case studies highlighted close associations between affective, cognitive, and behavioral components throughout the engagement process. Similar patterns of engagement were generated from the case studies, but these patterns differed from the literature-based hypothesized model in the order of influence of cognitive and affective engagement.

**Conclusion:**

Understanding how different components of engagement interact is essential for designing interventions that mitigate barriers to engagement and maximize intervention impact. The framework provides a preliminary guide and recommendations for how to support particular components. Future research on the order of cognitive and affective components (or importance thereof) and testing the influence of particular features on engagement components could improve the framework and clinical impact.

**Trial Registration:**

clinicaltrials.gov Identifier: NoObesity: NCT05261555; Wysa: NCT05533190; Dora R1: NCT05213390

Demand for health care far exceeds available supply. Face-to-face appointments are increasingly difficult to secure, and many individuals are going without needed care. Digital health interventions (DHIs) offer a promising solution to improve accessibility and efficiency.^1^, ^2^, ^3^, ^4^, ^5^ Supporting sufficient engagement is a common digital health challenge,^6^, ^7^, ^8^, ^9^, ^10^, ^11^, ^12^ but could increase interventions’ impacts on behavioral and clinical outcomes.^10^^,^^13^, ^14^, ^15^, ^16^, ^17^ Despite efforts to conceptualize and evaluate engagement, our understanding of the process of engagement with DHIs remains incomplete.^18^^,^^19^ Improving this understanding is necessary to mitigate barriers to engagement and to achieve optimum behavioral and health outcomes.^18^

Our understanding is complicated by varying conceptualizations of engagement.^1^^,^^19^, ^20^, ^21^, ^22^, ^23^ Though engagement components have no well-established definitions,^24^^,^^25^ they are broadly divided into 2 aspects: what users engage with (intervention—micro, health behavior—macro) and how they engage with it (affective, cognitive, and behavioral).^1^^,^^19^^,^^20^^,^^22^ Most DHI evaluations focus on behavioral engagement (captured by system use data),^1^^,^^19^^,^^26^, ^27^, ^28^ which hinders efforts to understand engagement as dynamic and multifaceted and to synthesize insights across studies. Definitions used in this study are based on previous literature (Supplemental Table 1, available online at http://www.mcpiqojournal.org).

Previous research has proposed models of how affective, cognitive, and behavioral responses proceed,^23^^,^^29^, ^30^, ^31^, ^32^ which informed hypothesis generation (Supplemental Table 2, available online at http://www.mcpiqojournal.org). Simplified diagrams illustrating the key proposed relationships between components in the models (Supplemental Figure 1, available online at http://www.mcpiqojournal.org) were synthesized in the context of digital health engagement to hypothesize a model (Figure 1).

**Figure 1:**
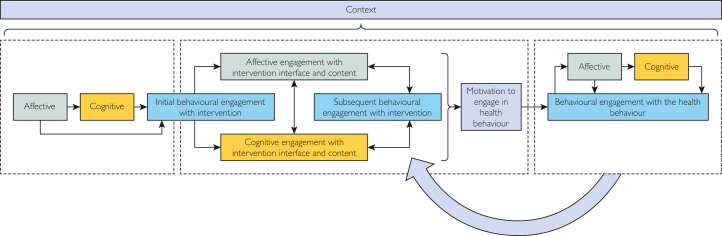
Hypothesized model of the process of engagement with a DHI and its target behavior (developed based on,^23^^,^^29^**^-^**^32^Supplemental Figure 1).

DHIs are used in a variety of clinical contexts. This analysis focused on DHIs applied in 3 areas: childhood obesity, mental health, and postsurgical follow-up. These DHIs address 2 interconnected needs: patient health outcomes and clinical burden. Childhood obesity and mental health are some of the most common health challenges of the 21st century.^33^, ^34^, ^35^ They can have considerable negative impacts on physical and mental health, quality of life, health care resources, and economies. Their prevalence means that there is a lack of capacity to provide support to all individuals who could benefit from it. Although both are complex conditions with many determinants, providing behavioral support can help improve patient outcomes.^36^^,^^37^ The third DHI aimed to improve patient outcomes by increasing the efficiency of postsurgical review. By automating routine tasks, this type of DHI can reduce clinician time spent on low-skill tasks and enable prioritization of patients with the greatest need. Together, these diverse DHI cases enable analysis of how engagement might vary in different contexts.

This study aimed to map the relationships between different engagement components in different types of DHIs. To achieve this aim, the key objectives were to (1) generate patterns of associations between engagement components; (2) compare patterns across different types of DHI to determine if identified processes are generalizable; and (3) provide evidence-based recommendations for strategies to optimize engagement.

## Methods

### Study Design

An exploratory multiple case study design following Yin’s procedures (Supplemental Figure 2, available online at http://www.mcpiqojournal.org) was used to conduct an in-depth exploration of cases in their real-world context.^38^, ^39^, ^40^, ^41^ Examining multiple cases strengthened the analysis by enabling replication, an assessment of transferability, and cross-case analyses to develop a framework of patterns of association between different engagement components.^41^ The standards for reporting qualitative research checklist^42^ was used to ensure completeness of reporting (Supplemental Appendix 1, available online at http://www.mcpiqojournal.org).

### Case Study Overview and Data Collection

Three case studies were selected for inclusion (Table 1). Engagement was examined primarily through thematic analysis of interview data, triangulated with questionnaire and system use data.^43^, ^44^, ^45^, ^46^ Both case studies 2 and 3 structured interview topic guides on the theoretical framework of acceptability.^47^ Ethical approval was obtained from the University of Oxford Medical Sciences interdivisional research ethics committee (REC) (R62092/RE001) and the University of Plymouth’s faculty research ethics and integrity committee (19/20-1316) for case study 1, the London - Stanmore REC (22/PR/0467) for case study 2, and the Health Research Authority (21/PR/0767) and the University of Plymouth’s faculty research ethics and integrity committee (2863) for case study 3.

**Table 1:** Characteristics of the Case Studies and Their Digital Health Interventions^a^

Intervention Characteristics	Case 1^43^^,^^44^^,^	Case 2 (Meinert et al, unpublished data, 2024)	Case 3^45^^,^^46^
Name	NoObesity	Wysa	Dora R1
Clinical area	Childhood obesity	Mental health	Ophthalmology (cataract surgery)
Intervention type	Mobile application	Mobile application	Telephone call
Conversational agent	No	Yes	Yes
Purpose	Behavior change	Behavior change	Clinical assessment
Intervention aim	Increase positive weight-related health behaviors (physical activity and diet)	Build mental resilience skills	Conduct postsurgical follow-up screening to improve clinical efficiency
Duration of use	Extended	Extended	One-time

Study characteristics	Case 1^43^^,^^44^	Case 2 (Meinert et al, unpublished data, 2024)	Case 3^45^^,^^46^
Study design	Mixed-methods (based on a Phase 1 implementation science approach).^38^^,^^39^	Mixed-methods randomized controlled trial (Meinert et al, unpublished data, 2024)	Mixed-methods (based on a Phase 2 implementation science approach),^38^^,^^39^ compared Dora R1 and supervising clinician decisions on symptoms and discharge recommendations
Research aims	Identify barriers and facilitators to users’ engagement and evaluate NoObesity’s impact on perceived motivation, self-efficacy, and health behaviors	Efficacy of Wysa at reducing symptoms of depression and anxiety compared with standard talking therapies care (waitlist control)	Evaluate Dora R1’s accuracy, safety, acceptability, usability, and potential for adoption into routine clinical care
Follow-up period	6 mo	3 mo	3 mo
Data collection methods	Semi-structured interviews, questionnaires, and application use data	Semi-structured interviews, validated questionnaires, application use data	Semi-structured interviews, validated questionnaires, system and clinician decision data
Qualitative sample size	n=15	n=4	n=20
Quantitative sample size	n=35	n=46 (survey) n=30 (application data)	n=202 (system data) n=180 (survey)

a Full details published elsewhere.^43^^,^^45^

### Data Analysis

#### Within*-*Case Analyses

A codebook thematic analysis approach^48^^,^^49^ was used to analyze qualitative data. Original case study analyses did not specifically examine patterns of associations between engagement components, so additional analyses were conducted to generate mappings. The initial thematic frameworks and transcripts were re-evaluated through the lens of the multifaceted conceptualization of engagement, using the definitions in Supplemental Table 1. The relationships between components were described in terms of patterns of influence (eg, initial micro behavioral engagement → micro affective engagement → subsequent micro behavioral engagement). These patterns were represented in diagrams summarizing all coded relationships.

#### Cross-Case Analysis

Comparability was examined to determine whether synthesis across cases was valid by identifying the key differences and considering how they might influence the relationships between engagement components. The cross-case analysis used 3 techniques: pattern matching, cross-case synthesis, and explanation building (or hypothesis generating).^41^ Pattern matching compared within-case empirically-based models to each other and the hypothesi*z*ed model.^41^ Cross-case synthesis brought together within-case findings and explored similarities and differences.^41^^,^^50^^,^^51^ Explanation building was applied to aggregate findings and develop a refined model. Alternative explanations were generated and explored in the context of the data.^41^^,^^52^ The refined conceptual model, positing how the different components of engagement influence each other and what factors can mediate these relationships, was combined with a previous systematic review^53^ to recommend strategies for supporting engagement with DHIs.

## Results

### Within-Case Findings

The full case study results are published elsewhere (Meinert et al, unpublished data, 2024)^43^^,^^45^; study characteristics, key findings, and within-case mappings are summarized here to contextualize the cross-case synthesis.

#### Case Study 1: NoObesity

The initial analysis explored factors associated with families’ mobile application engagement.^43^ Key facilitators included novelty, interactivity, supported goal setting and self-monitoring, positively-framed content, feedback, and reminders. Boredom and forgetting to use were key barriers, as were contextual factors that shaped families’ opportunities, motivation, and ability to engage with target behaviors (eg, weather, time, accessible and affordable resources, and children’s age).^43^

**Figure 2:**
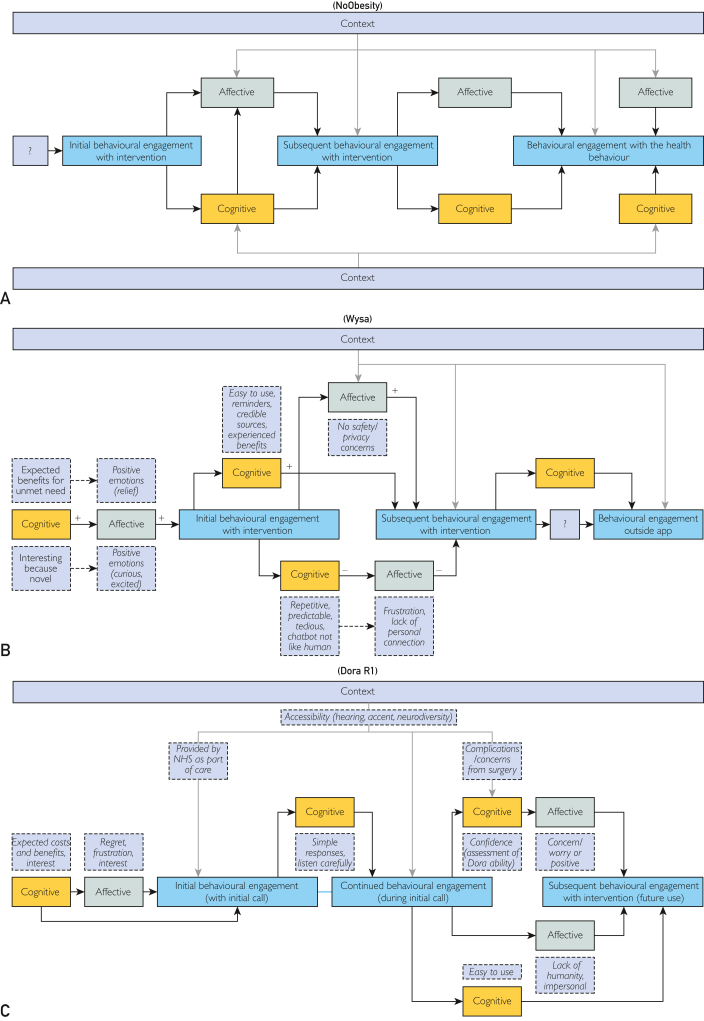
Mapping of engagement components derived from NoObesity (A), Wysa (B), and Dora R1 (C) qualitative data (coded against the thematic frameworks in Supplemental Tables 3-5).

No data was captured about participants’ cognitive or affective engagement with the idea of the application before use; component mapping began after initial behavioral engagement. Behavioral engagement with the intervention shaped affective and cognitive engagement with it, which in turn influenced subsequent micro and macro engagement (Figure 2A). Affective and cognitive components were too integrated to discern their direction of influence on each other. Engagement components could have positive or negative influences on one another. For example, positive affective engagement with the mobile application’s aesthetic design and tone encouraged subsequent behavioral engagement, whereas feeling uncomfortable about tracking children’s weight or being cognitively engaged by poorly-timed or too frequent notifications decreased motivation to behaviorally engage with the application (Supplemental Table 3, available online at http://www.mcpiqojournal.org).

#### Case Study 2: Wysa

The randomized control trial aimed to evaluate Wysa’s efficacy at improving depression and anxiety symptoms compared with a waitlist control, but low sample size prevented reliable statistical analysis (Meinert et al, unpublished data, 2024). Positive cognitive engagement with the idea of the mobile application was associated with positive affective engagement and initial behavioral engagement (Figure 2B, Supplemental Table 4, available online at http://www.mcpiqojournal.org). Micro and macro engagement overlapped because engagement with target behaviors (eg, thought monitoring and meditation exercises) primarily occurred through the application. Subsequent behavioral engagement was also influenced by cognitive engagement via affective engagement, although both cognitive and affective components could also influence subsequent microbehavioral engagement independently. Engagement was hampered by limitations of the conversational agent (CA), which caused frustration and reduced motivation to use Wysa (Meinert et al, unpublished data, 2024). The variety of content influenced cognitive engagement; it was viewed as helpful but overwhelming to navigate (Meinert et al, unpublished data, 2024). There was little evidence to suggest that engagement with the intervention supported engagement with the therapeutic techniques beyond the mobile application (Meinert et al, unpublished data, 2024).

#### Case Study 3: Dora R1

The initial analysis aimed to assess Dora R1’s accuracy, safety, acceptability, and usability for (primarily older) patients.^45^ Unlike NoObesity and Wysa, which were longer-term behavioral interventions, Dora R1 was a brief clinical assessment, so the study focused on micro engagement. Contextual factors (eg, provision of call through the UK’s National Health Service and postsurgical complications) had an important influence on engagement (Figure 2C, Supplemental Table 5, available online at http://www.mcpiqojournal.org). Although the call was generally perceived as acceptable and potentially beneficial*,*^45^ the lack of human element and concerns about the CA’s capacity to understand nuances in patient responses influenced affective and cognitive engagement. As with Wysa, the analysis indicated a pattern of cognitive engagement influencing behavioral engagement through affective engagement, although cognitive and affective engagement could also influence behavioral engagement independently.

### Comparability

Key differences that could influence case comparability included clinical application, intervention type and aim, and study methodology. Clinical application was expected to shape the importance or direction of components’ influences, rather than the pattern of how they interacted. In terms of intervention type and aim, the first 2 cases represented similar interventions (prolonged mobile application-based behavior change interventions), whereas the third was different (brief telephone assessment). These cases enabled a comparison of interventions that were expected to produce similar patterns of engagement and those that would be expected to produce a predictably different pattern.^41^ Non-behavior change DHIs still require micro engagement to achieve their intended purpose; the relative importance of components might differ, but this does not imply that the structural pattern of interactions should vary. Therefore, cases 1 and 2 should be comparable to case 3 for, but not beyond, micro engagement. In terms of methodology, although all case studies qualitatively examined acceptability and engagement, interview structures evolved over their sequential execution. Re-evaluation of the original data and thematic frameworks aligned within-case engagement mappings around the same research question; however, the extent to which the whole process of engagement could be examined for each case was limited by available data.

### Cross-Case Findings

All the models reported that affective and cognitive engagement influence and are influenced by behavioral engagement. In this way, empirical results supported the literature-based hypothesis of close interactions between these components. Empirically-generated relationships between engagement components were largely similar, despite differences between the cases, but differed from the hypothesized mapping in the order of influence of affective and cognitive engagement. The case study analyses did not support the hypothesized pattern of affective engagement influencing behavioral engagement via cognitive engagement.^23^^,^^29^, ^30^, ^31^, ^32^ The opposite pattern was observed: cognitive engagement influenced behavioral engagement via affective engagement (Supplemental Figure 3, available online at http://www.mcpiqojournal.org).

Supplemental Table 6 (available online at http://www.mcpiqojournal.org) details all the patterns of engagement to facilitate comparison and interpretation of how and why particular engagement components are related, what that means in the context of the cases, and what broader insights for engagement with digital health can be generated. Neither Supplemental Table 6 nor Supplemental Figure 3 should be interpreted as a numeric tally indicating the strength of particular patterns; there are insufficient cases for such a tally to be meaningful.^41^ Table 2 summarizes the hypotheses and the extent to which they are supported by the findings.

**Table 2:** Summary of Hypotheses and Supporting Evidence

Hypothesis	Evidence From the Cross-Case Analysis
Initial micro engagement (case studies 2 and 3)	
H1a: Given that an individual is aware of and able to access a digital health intervention, initial micro behavioral engagement will be driven by affective engagement with the idea of the intervention either directly or indirectly by influencing cognitive engagement (the individual’s appraisal of the expected benefits, risks, and demands of intervention).	The analysis supported the hypothesis that initial behavioral engagement was driven by both affective and cognitive engagement; however, the patterns of engagement developed from the data in both case studies suggested that cognitive engagement influenced behavioral engagement via affective engagement, rather than affective engagement influencing behavioral engagement via cognitive engagement as hypothesized. The key finding was that both affective and cognitive engagement could influence initial behavioral engagement; determining the order of influence was vulnerable to bias in reporting and interpretation.
H1b: Both positively-valenced and negatively-valenced emotions can support affective engagement and initial micro behavioral engagement.	There was evidence for positively-valenced affective engagement supporting initial behavioral engagement across the studies. In both cases, curiosity and interest were reported as encouraging initial engagement; whether these can be considered emotions is debatable,^66^^,^^67^ but they have affective as well as cognitive aspects that make them relevant for the assessment of this hypothesis. Evidence for the influence of negatively-valenced emotions was more limited but aligned with H1b. Negative emotions (worry about a lack of mental health support) were associated with positive affective engagement (feelings of relief) and behavioral engagement with Wysa, supporting H1b. On the other hand, negative emotions expected by some Dora R1 users when communicating with a machine did not prevent their engagement. Such emotions may have contributed to other patients declining to participate, but only 5/542 eligible but not included patients specifically mentioned dislike of automation as their reason for nonparticipation.^45^
H1c: Only positive cognitive engagement will support initial micro behavioral engagement.	This hypothesis was supported but cannot be confirmed with the data available, as there were no findings suggesting that negative cognitive engagement (ie. lack of attention or interest, judgments that costs outweighed potential benefits, etc.) increased behavioral engagement. Negative cognitive engagement with the idea of a DHI would either be represented as a lack of attention, a lack of interest, or a judgment of the intervention as too difficult to use or not likely to be of sufficient benefit for the effort or costs required. An association between cognitive disengagement and behavioral engagement would not align with theoretical expectations.
Subsequent micro engagement (case studies 1, 2, and 3)	
H2a: An individual’s initial behavioral engagement with a digital health intervention will influence their affective and cognitive engagement with the intervention by providing further experiences, which generate subsequent affective responses and cognitive assessments.	This hypothesis was supported by evidence across the studies indicating that participants’ use of a DHI shaped their subsequent affective, cognitive, and behavioral engagement with it. As with H1a, the main difference between the hypothesized and empirical mappings was that, in contrast to the hypothesized order (affective → cognitive), the pattern generated from all 3 case studies when there was an indirect relationship was cognitive engagement influencing affective engagement.
H2b: The intervention needs to be sufficiently affectively OR cognitively engaging to maintain behavioral engagement with the intervention interface and content.	This hypothesis was somewhat supported by the findings. Themes from case studies 1 and 2 indicated that both affective and cognitive disengagement could increase the likelihood of behavioral disengagement. For example, once all content in the NoObesity application had been explored, users became bored and experienced less motivation to use the application. Similarly, Wysa’s CA limitations led to frustration and decreased motivation to engage. However, the influence of cognitive and affective engagement could not be sufficiently distinguished to determine if one alone would be sufficient to sustain behavioral engagement. In case study 3, the role of context was particularly important. For example, most participants completed Dora R1’s brief intervention, regardless of their attitudes toward it. This suggests that brief DHIs with a single clear objective may not need to be especially interesting or enjoyable to support the limited behavioral engagement required.
Translation of micro to macro engagement (case studies 1 and 2)	
H3a: Affective or cognitive engagement with the intervention content will increase an individual’s motivation to engage in the target health behavior.	This hypothesis was supported by findings from case studies 1 and 2 suggesting that affective, cognitive, and behavioral engagement with the intervention were associated with engagement with the target behavior. For example, parents reported that cognitively engaging with content that highlighted the health benefits for children would increase their motivation to do the behavior and that framing the behaviors as a family activity made their perceptions more positive. Although engagement with the intervention and behavior were conflated in Wysa because users were guided through therapeutic exercises on the application, the same pattern was observed (eg, positive experiences encouraged further engagement with the in-application therapeutic exercises).
H3b: The translation of motivation into behavior is dependent on sustained cognitive engagement (attention) and influenced by the individual’s context.	All findings highlighted the importance of reminders for prompting DHI engagement, but attention could not be specifically linked to motivation with the available evidence. Context played a role across the process of engagement in all cases and was associated with motivation, primarily in case study 1. Themes from both NoObesity and Wysa highlighted that lack of attention was a key barrier to subsequent engagement; reminders were an important facilitator to help prompt micro and macro engagement.
Macro engagement and influence on subsequent micro engagement (case studies 1 and 2)	
H4a-c	There was insufficient evidence to determine whether these hypotheses could be supported.

**Figure 3:**
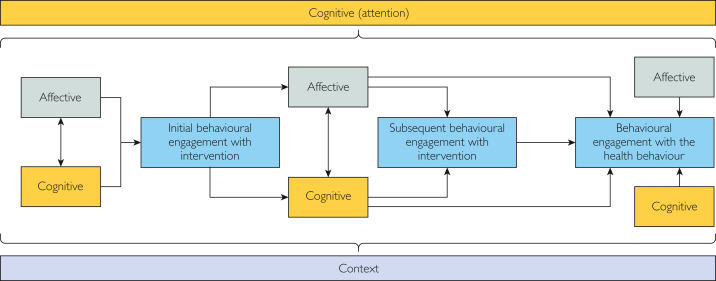
Proposed model of relationships over the process of affective, cognitive, and behavioral engagement with digital health interventions.

The hypothesized model and empirical models were refined into a new model (Figure 3). Although this model does not answer the question of the order of influence of affective and cognitive engagement (given the disparity in the literature and case study findings), it represents an important step in integrating empirical evidence into existing theory.

## Discussion

### Principal Findings

The cross-case analysis highlighted the importance and interconnectedness of affective, cognitive, and behavioral engagement. Patterns generated from the case studies were largely similar; the main difference between hypothesized and empirical models was the order of influence of cognitive and affective engagement on behavioral engagement. The hypothesized model posited that affective engagement influenced cognitive engagement, whereas the opposite trend was observed in the case studies.

### Potential Explanations for Differences Between Hypothesized and Empirical Mappings

One potential explanation is that the hypothesized model was developed from various models (Supplemental Figure 1). Only one was developed from an evaluation of engagement with a DHI,^30^ although it shared the same hypothesized affective → cognitive pattern as the others. Alternatively, given the established relationship between affective and cognitive processes,^54^^,^^55^ there could be an ongoing feedback loop that is difficult to disentangle. Our analysis may have captured different stages of this loop when compared with previous studies. For example, in case study 2, participants were worried about a lack of mental health support while on a waitlist. It could be argued that this represents affective engagement, which leads to cognitive engagement (consideration and selection of options to address this negative emotional state). Although this worry does represent an affective state, it would not be considered affective engagement under our definition, which is specific to perceptions of and experiences engaging with the intervention and behavioral goal (Supplemental Table 1). The difference could also be because of methodology. Retrospective, qualitative data is limited by recall and shaped by participants’ subsequent interpretation. They may have experienced affective engagement before cognitive engagement, but been unaware of it,^29^ or ordered their recollections in a way that made more logical sense to convey (eg, “I felt X because Y” would reflect the cognitive → affective pattern generated in our analysis).

### Strengths and Limitations

The use of a multiple case study design, rather than a single case study design, was a strength, as it enabled an examination of the transferability of findings.^56^ The aim of case study research is not to achieve statistical generalisability^41^ but to generate insights to shape theoretical propositions about how users engage with DHIs. Considering similarities and differences between cases enabled hypotheses to be generated about how these findings might apply to a broader model of digital health engagement. The posited framework is intended as a hypothesis—further testing will be required to confirm or refine proposed associations.

The main limitation of the cross-case analysis is the scope of comparability. The differences in DHI were deliberate, to enable an exploration of how DHI characteristics might influence engagement. However, differences in the DHIs and methodologies meant that the whole engagement process could not be compared across all cases. Case study 1 semi-structured interviews did not capture factors influencing initial engagement (H1a-c); case studies 2 and 3 were designed to address this limitation, but the synthesis would have been stronger with data from all 3 studies. As there was no health behavior engagement with Dora R1, the analysis of H3a-b and H4a-c was limited to case studies 1 and 2.

The individual case study thematic analyses were conducted by multiple reviewers, but due to resource constraints, the cross-case synthesis was only conducted by 1 researcher. To mitigate this limitation, the cross-case analysis was reconducted after a couple months to identify and challenge assumptions.

The sample size in case study 2 (Wysa) was smaller than anticipated, which limited the within-case analysis. Our thematic analysis indicated CA limitations influenced affective, cognitive, and behavioral engagement. Previous evaluations of Wysa have reported more positive evidence of engagement (median user retention=51 days,^57^ weekly engagement rate=57%,^58^ and more expressions of gratitude (n=212) than dissatisfaction (n=15) toward the CA^59^); however, differences in levels of engagement likely would not alter the relational patterns between engagement components.

### Comparison to Previous Research and Theoretical Implications

Recent research has aimed to clarify definitions of engagement components,^18^^,^^25^ with a couple key differences from Supplemental Table 1. Bijkerk et al^25^ excluded the concept of motivation, which we included to ensure it was captured in the analysis. Kelders et al^18^ definition of behavioral engagement included effort to use, which we included in cognitive engagement (Milne-Ives et al, unpublished data, 2025). Although similarities between definitions indicate that we are moving toward consensus, differences highlight the need for transparency in reporting, iterative efforts toward conceptual clarity (Milne-Ives et al, unpublished data, 2025), and further research on how interactions between components of engagement can shape DHI design.^18^ Our paper outlines a theory- and evidence-based framework of how the different components of engagement relate to each other and shape the overall process of engagement with DHIs. The proposed framework provides guidance for conceptualizing and evaluating the different components of engagement in future research.

### Implications for DHI Developers

An improved understanding of interactions between engagement components will enable DHI design to target particular and personalized barriers to engagement, thereby improving impact on behavioral, health, and other outcomes. The analysis evidenced the ability of particular design features and Behavior Change Techniques to support engagement with DHIs at different components and stages (Supplemental Figure 4, available online at http://www.mcpiqojournal.org, demonstrates where in our model these features are posited to influence the different components). Supplemental Table 7 (available online at http://www.mcpiqojournal.org) integrates cross-case analysis findings with our previous systematic review^53^ to provide recommendations for designing interventions that support engagement and avoid key barriers. This is not exhaustive, but includes general recommendations and cautions as a foundation for further work (aligned with recent literature).^22^^,^^59^, ^60^, ^61^, ^62^ The needs, preferences, and engagement styles of individuals will vary^18^ and should be explored through user-centered design.

### Implications for Clinical Care

There are important implications for clinical care: if patients engage more effectively with DHIs, we would expect to see improved behavioral and clinical outcomes. Although some studies support this, there is a lack of consistent evidence of a strong, positive association between engagement and clinical outcomes, hindered by limitations in digital health engagement evaluations.^10^^,^^13^, ^14^, ^15^, ^16^, ^17^ Advancing our theoretical understanding is critical to enable future research to examine this relationship with more clarity and consistency.

Empowering patients to take greater responsibility for their health has been associated with increased self-efficacy, which has in turn been associated with increased motivation and behavioral adherence.^63^ Therefore, we can hypothesize that supporting engagement will increase the likelihood that an intervention will achieve its outcomes. If so, this would likely snowball into other positive implications for clinical care; if patients are better able to self-manage their health and engage in preventive health behaviors, there may be a decrease in clinical burden and a freeing of clinical resources to focus on patients with more complex conditions. Similarily, adoption and engagement with artificial intelligence-based clinical interventions could improve clinical efficiency by reducing clinical burden and enabling clinicians to engage more with high-skill tasks that have greater patient benefits.

## Conclusion

Supporting engagement with DHIs can help address the gap between clinical resources and demand, with valuable implications for public health and clinical care.^64^^,^^65^ While it is accepted that engagement is multifaceted,^19^, ^20^, ^21^, ^22^, ^23^ little is known about how different components interact and how best to design interventions to address barriers in particular stages of the engagement process.^18^ This cross-case analysis investigated the generalizability and factors influencing patterns of engagement with 3 DHIs.^41^^,^^66^ Despite the close relationships between all components (especially affective and cognitive engagement), there is value in the process of trying to disentangle them.^67^^,^^68^ The model proposed here provides a prompt for intervention developers to consider how users will engage with their intervention and how to incorporate strategies to support engagement. Developers can use our framework to identify barriers, what component(s) of engagement they are affecting, and how they could best be mitigated. Future research could further advance our understanding by testing and refining this framework with other DHIs, exploring whether it is meaningful to identify the order of components, and evaluating how other factors, features, and contexts might influence components over the process of engagement. Ultimately, this improved understanding is necessary to optimize intervention design.

## Potential Competing Interests

Professor Meinert reports financial support was provided by the South East School of Public Health, Workforce Training and Education Directorate, NHS England and by the National Institute for Health Research (NIHR) and NHSX. Dr Milne-Ives reports financial support was provided by the South East School of Public Health, Workforce Training and Education Directorate, NHS England and by the NIHR and NHSX. All other authors declare no financial or non-financial competing interests.

## Ethics Statement

Ethical approval was obtained from the University of Oxford Medical Sciences Interdivisional Research Ethics Committee (R62092/RE001) and the University of Plymouth’s Faculty Research Ethics and Integrity Committee (19/20-1316) for case study 1, the London - Stanmore Research Ethics Committee (22/PR/0467) for case study 2, and the Health Research Authority (21/PR/0767) and the University of Plymouth’s Faculty Research Ethics and Integrity Committee (2863) for case study 3. Informed consent was obtained from all participants in the 3 case studies.
