# Supplementary figures and images for: Mapping the Process of Engagement With Digital Health Interventions: A Cross-Case Synthesis
Source: Mayo Clin Proc Innov Qual Outcomes. 2025 May 27;9(3):100625. doi: 10.1016/j.mayocpiqo.2025.100625 (PMC12158608; doi:10.1016/j.mayocpiqo.2025.100625)

## Supplemental Figure 2. Procedures of the multiple case study approach<sup>52</sup>

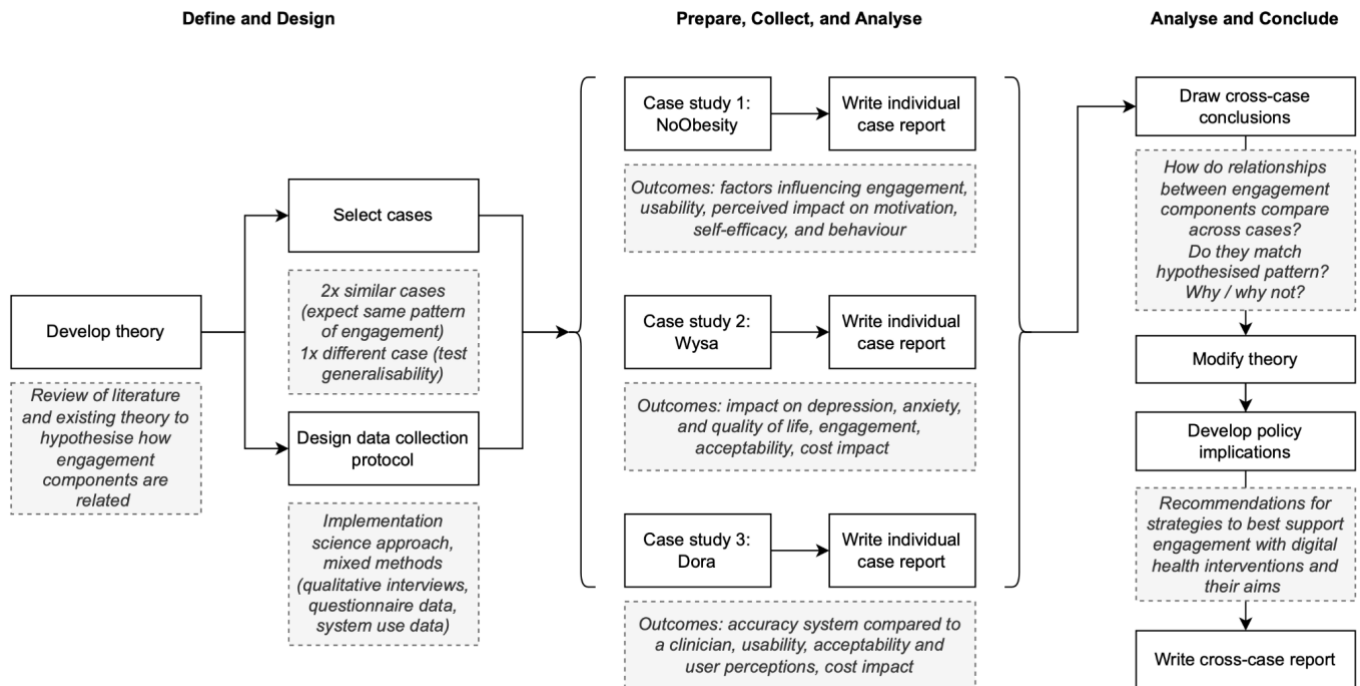

Supplement: Supplemental Figure 2 [file mmc3.pdf]
