# Supplementary material for: Mapping the Process of Engagement With Digital Health Interventions: A Cross-Case Synthesis
Source: Mayo Clin Proc Innov Qual Outcomes. 2025 May 27;9(3):100625. doi: 10.1016/j.mayocpiqo.2025.100625 (PMC12158608; doi:10.1016/j.mayocpiqo.2025.100625)
Supplement: Supplemental Figure 3 [file mmc4.pdf]

Supplemental Figure 3. Combined mappings of NoObesity, Wysa, and Dora R1

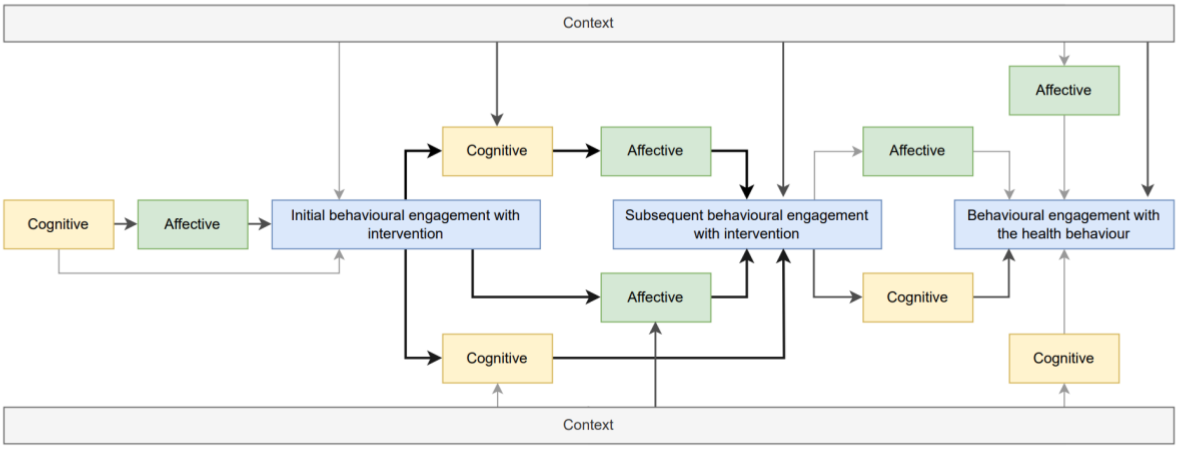

(Note: the colour and weight of the arrows indicates whether a particular relationship was generated in 1, 2, or 3 cases; this is for illustrative purposes only, it does not represent a statistical count of evidence)
