# Supplementary material for: Mapping the Process of Engagement With Digital Health Interventions: A Cross-Case Synthesis
Source: Mayo Clin Proc Innov Qual Outcomes. 2025 May 27;9(3):100625. doi: 10.1016/j.mayocpiqo.2025.100625 (PMC12158608; doi:10.1016/j.mayocpiqo.2025.100625)

Supplemental Figure 4. Case study-based hypotheses for how various factors can influence the relationships between different components of engagement

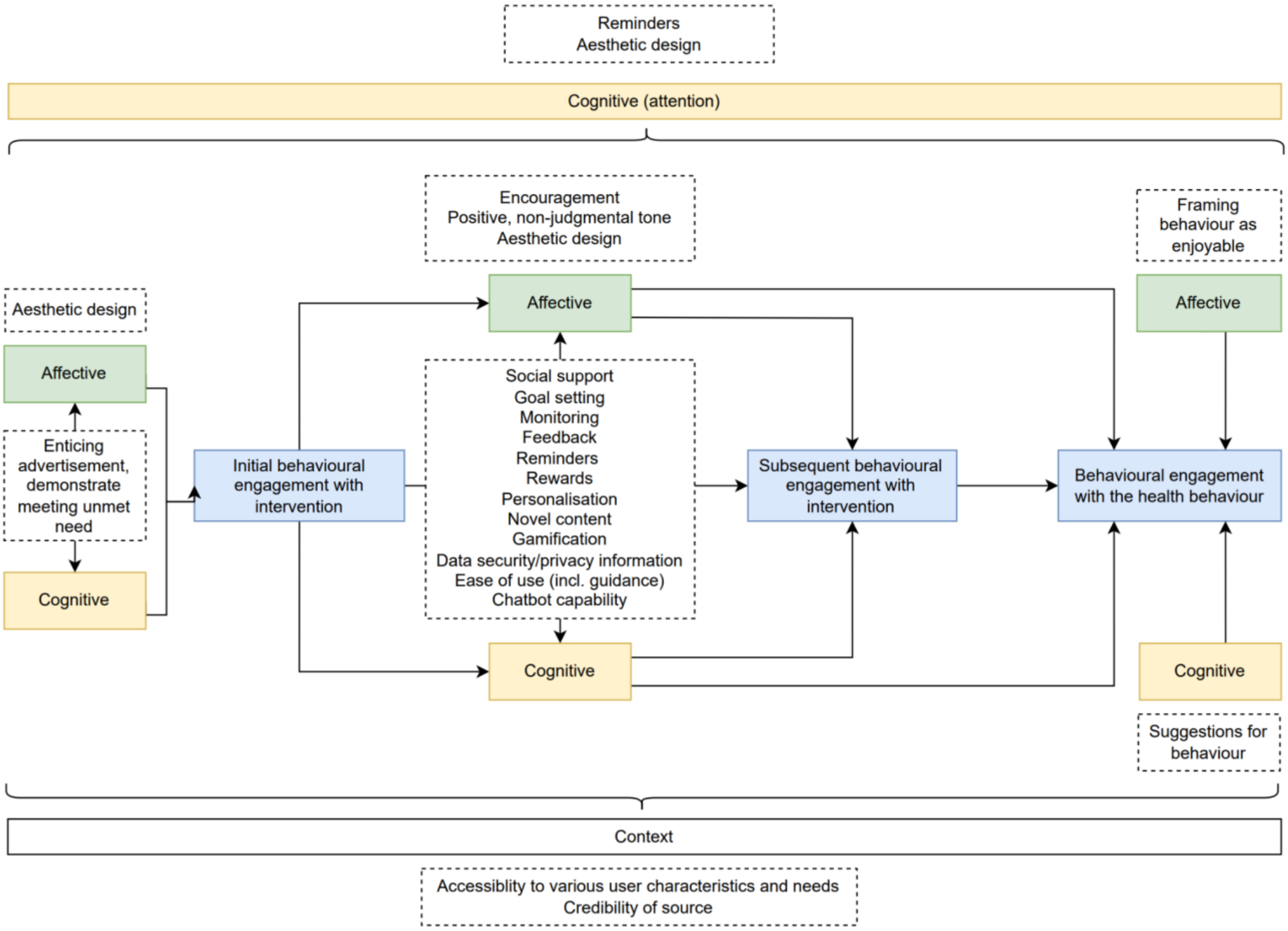

Supplement: Supplemental Figure 4 [file mmc5.pdf]
