# Supplementary material for: Mapping the Process of Engagement With Digital Health Interventions: A Cross-Case Synthesis
Source: Mayo Clin Proc Innov Qual Outcomes. 2025 May 27;9(3):100625. doi: 10.1016/j.mayocpiqo.2025.100625 (PMC12158608; doi:10.1016/j.mayocpiqo.2025.100625)
Supplement: Supplemental Table 1 [file mmc6.pdf]

Supplemental Table 1. Definitions of key terms

Term	Definition
Affective engagement	The positive and negative feelings, emotions, moods, interest, and attitudes (e.g. enjoyment, satisfaction, frustration) related to perceptions of and experiences engaging with the intervention and the behavioural goal[20,24,32–36]; including elements of motivation relating to the alignment of the intervention and target behaviour to users' goals and values, in both the immediate moment and in the long-term[34,37–40].
Behavioural engagement	Individuals' interaction with a stimulus or their participation in or performance of a particular activity. This can include quantity and quality of use - frequency, amount, depth, duration, and accordance with intended use in line with intervention goal - of the intervention and performance of the target health behaviour[20,23,24,36,41].
Cognitive engagement	Users' interest and attentional effort invested in the decision-making processes and learning associated with the intervention and the target behaviour[23,24,33,36,37]; including elements of motivation relating to assessments of capability, risks, and benefits (perceived usefulness, control, and potential for success)[32,34,35,40].
Micro engagement	"Moment-to-moment engagement with the intervention"[2]; defined for this study as engagement with the digital health device, interface, and content (active intervention ingredients)[21]
Macro engagement	Including "engagement and identification with the wider intervention goals"[2] and "engagement with the targeted health behaviour"[21]
