# Supplementary material for: Mapping the Process of Engagement With Digital Health Interventions: A Cross-Case Synthesis
Source: Mayo Clin Proc Innov Qual Outcomes. 2025 May 27;9(3):100625. doi: 10.1016/j.mayocpiqo.2025.100625 (PMC12158608; doi:10.1016/j.mayocpiqo.2025.100625)
Supplement: Supplemental Table 2 [file mmc7.pdf]

Supplemental Table 2. Hypotheses regarding patterns of relationships between engagement components

**Stage of engagement**	**#**	**Hypothesis**
Initial micro engagement with the DHI	1a	Given that an individual is aware of and able to access a digital health intervention, initial micro behavioural engagement will be driven by affective engagement with the idea of the intervention either directly or indirectly by influencing cognitive engagement (the individual's appraisal of the expected benefits, risks, and demands of intervention).
	1b	Both positively- and negatively-valenced emotions can support affective engagement and initial micro behavioural engagement.
	1c	Only positive cognitive engagement will support initial micro behavioural engagement.
Subsequent micro engagement with the DHI	2a	An individual's initial behavioural engagement with a digital health intervention will influence their affective and cognitive engagement with the intervention by providing further experiences which generate subsequent affective responses and cognitive assessments.
	2b	The intervention needs to be sufficiently affectively OR cognitively engaging to maintain behavioural engagement with the intervention interface and content.
Translation of micro engagement with the DHI to macro engagement with the behaviour	3a	Affective or cognitive engagement with the intervention content will increase an individual's motivation to engage in the target health behaviour.
	3b	The translation of motivation into behaviour is dependent on sustained cognitive engagement (attention) and influenced by the individual's context.
Macro engagement with the behaviour and influence on subsequent micro engagement with the intervention	4a	As the individual engages with the health behaviour, affective, cognitive, and behavioural components of engagement with the health behaviour will all influence each other to determine whether macro behavioural engagement is sustained.
	4b	High macro engagement with the health behaviour may result in decreased micro engagement with the intervention as the health behaviour becomes more habitual.
	4c	Decreasing macro engagement with the health behaviour may prompt an increase in micro engagement with the intervention, if there is still macro affective or cognitive engagement with the health behaviour goal.
