# Supplementary material for: Mapping the Process of Engagement With Digital Health Interventions: A Cross-Case Synthesis
Source: Mayo Clin Proc Innov Qual Outcomes. 2025 May 27;9(3):100625. doi: 10.1016/j.mayocpiqo.2025.100625 (PMC12158608; doi:10.1016/j.mayocpiqo.2025.100625)
Supplement: Supplemental Table 3 [file mmc8.pdf]

Supplemental Table 3. Case Study 1: Thematic framework mapping factors to engagement components and patterns of engagement

Factor	Factor association	COM-B component	TDF	Ppt	Quotes	Eng. component	Pattern of engagement
Engagement							
Aesthetic appeal	Positive	Motivation (auto)	Emotion	5<br><br>4	I didn't find the colors, the green, I didn't find it appealing. Like, there are some apps that I go on because they just appeal to me and I just find the colors vibrant and I just enjoy being on the app. This app, I didn't find that. ... Aesthetically, it was not pleasing to... If I was in a horrible mood, it didn't make me in a better mood ... it's just a very depressing app<br><br>Yeah. I think it looks really good. It looks engaging. You know, if you show this to a child, they would find it interesting because of the iconography and how it looks.	Affective<br><br>Cognitive<br><br>Behavioural	Initial Behavioural (micro) → Affective → Subsequent Behavioural (micro)<br><br>Initial Behavioural (micro) → Cognitive → Subsequent Behavioural (micro)
Discomfort tracking weight	Negative	Motivation (auto)<br>Motivation (ref)	Emotion<br><br>Beliefs consq.	10<br><br>12	I don't think my daughter, who is eight, would have liked seeing, if she had been on the slightly heavier side or whatever, I don't think she would have liked seeing it. I think she is getting to an age where she is more aware of it.<br><br>I am thinking as me as a parent, I didn't want to put the details in there and be too hung up on measurement, if you know what I mean.	Affective<br><br>Cognitive<br><br>Behavioural	Initial Behavioural (micro) → Affective → Subsequent Behavioural (micro)<br><br>Initial Behavioural (micro) → Cognitive → Subsequent Behavioural (micro)
Feedback / visualisations	Positive	Capability (psych)<br>Motivation (auto)	Reinforce-ment<br><br>Knowledge	4<br><br>5	I think the feedback you got in that family survey thing is helpful because it's equating it to what you're actually doing, what you might think, "Well, okay. I eat enough fruit in the day. Because you think you eat one apple." But obviously, if you put that in there, it's going to say, "Well, you're not eating enough fruit, so you need to think about it."<br><br>If there was a graph or something, I could see that it's declining, my water intake, it would make me want to act on that. But I couldn't see my progress... it was not visually engaging enough for me to think ooh, look, my graph's going down. I haven't been drinking as much water as I said I'd like to. Let me get back onto it.	Cognitive<br><br>Behavioural	Initial Behavioural (micro) → Cognitive → Subsequent Behavioural (micro)<br><br>Behavioural (micro) → Cognitive → Behavioural (macro)

Integration	Positive	Opportunity (phys)	Envr.<br><br>Social	1<br><br><br>4	If [my kids] could connect their smartwatch to that, and their step counts, and be in competition and that kind of thing. Yes, they would love it.<br><br>Obviously, you're having all your weight checks and that if they feel that there's a problem, that they will refer you to these healthy eating things. So that would really help, I suppose, at that point to click in and say, "Well, look, use this. We'll link it to them, and put it in at that point because that's trying to get it as early as possible.	Affective<br><br>Cognitive<br><br>Behavioural	Initial Behavioural (micro) → Affective → Subsequent Behavioural (micro)<br><br>Initial Behavioural (micro) → Cognitive → Subsequent Behavioural (micro)<br><br>Context → Cognitive → Behavioural (macro)
Interactivity / gamification	Positive	Motivation (auto)	Envr.<br><br>Emotion, Reinforce-ment<br><br>Goals<br><br>Social	14<br><br><br><br><br>9	It's like human nature, we all want to get these wee awards and get these trophies and earn these points, whether that's just a personality or whatever it is. I know that as soon as you set a goal on something, I'm going to want to achieve it and I'm going to want to do the best at it that I can.<br><br>Something to make it more interactive, more fun. I don't know, like daily challenges for the children or I don't know. You've now been eating fruits for a whole month consistently. You've unlocked 10 new character. It's more like game driven, but informative game driven.	Affective<br><br>Cognitive<br><br>Behavioural	Initial Behavioural (micro) → Affective → Subsequent Behavioural (micro)<br><br>Initial Behavioural (micro) → Cognitive → Subsequent Behavioural (micro)<br><br>Behavioural (micro) → Affective → Behavioural (macro)<br><br>Behavioural (micro) → Cognitive → Behavioural (macro)
Negative / positive connotations	Either	Motivation (auto)	Envr.<br><br>Emotion, Reinforce-ment	5<br><br><br><br><br><br><br>4<br><br><br>12	Again, I think that it could just have been a bit more encouraging. So if I went days, for example, without doing well, I'll just put it like that, it just said oh, success recorded or something like that, was coming up. If we'd [inaudible 00:06:49] badly, it could have been a bit more attractive, a bit more engaging. And I know that it's... technology, but I just think that it could offer a lot more, but it just wasn't offering it.<br><br>The word [obesity] is what it is and it's a word that is used. It's obviously got lots of negative connotations.<br><br>Possibly, in, for me, because I think that, everything's connected to how you feel about yourself, how you enjoy going out in the fresh air and the things that you're eating, they're all connected. So, for me, how you feel would have been better.	Affective<br><br>Behavioural	Initial Behavioural (micro) → Affective → Subsequent Behavioural (micro)<br><br>Affective → Behavioural (macro)

Notifications	Either	Capability (psych)<br>Motivation (auto)<br>Motivation (ref)	Memory...<br><br>Emotion	3<br><br><br><br><br>8	I think it's more the timing. I don't mind the notifications coming up, and if it was in the evening and it reminded me sort of when my little girl was in bed, then I probably would have gone on to the app, and ticked it off, and kept up with it. I think it was more the time in.<br><br>Not necessarily pestering you with notifications, like notifications would be good to remind you to log it. But if the feedback was just giving you in the app when it was open, I think that'd be really good because I think people are motivated by that	Affective<br><br>Cognitive<br><br>Behavioural	Initial Behavioural (micro) → Cognitive → Affective → Subsequent Behavioural (micro)<br><br>Initial Behavioural (micro) → Cognitive → Subsequent Behavioural (micro)
Novelty / variety	Positive	Capability (psych)<br>Motivation (auto)	Memory...<br><br>Emotion<br><br>Beliefs consq.<br><br>Envr.	2<br><br><br><br>15	It's very limited in what you can and cannot do with it. They've got a little bit of games, but after you've done that a couple of times, you're not bothered in it anymore.”<br><br>There was nothing new, there was no change to it. Actually, had there been some new content on there, it would have encouraged me to come back more and more and go, "Oh, what more can I learn? Can I beat it again on learning something else?"	Cognitive<br><br>Behavioural	Initial Behavioural (micro) → Cognitive → Subsequent Behavioural (micro)
Personalisation	Positive	Capability (psych)	Envr.	15	That was something else I really liked as well actually, the photo. To make it personalized to us. That was one of the first things I did to make it really like it was about us. So no, I did like that aspect as well, to be able to actually personalize it. I suppose that's the only other thing, if there was any other ways of personalizing it a little bit more to make it our family and things like that.	Affective<br><br>Behavioural	Initial Behavioural (micro) → Affective → Subsequent Behavioural (micro)
Ease of use	Positive	Capability (psych)	Memory...	9<br><br><br><br>3	It was very easy. It wasn't complicated. It wasn't something that I needed to fill out a lot of details or try to really use my brain to think, "Why is this not working?"<br><br>So, maybe it was just like a one click kind of thing for that day, or if it could come off as like a question or something, "Have you drank your water for today?" And, you can just click Yes, rather than going through and tick it on each day. Or, have to be in your three or four jog that you said you were going to do, and you could just click yes or no. That would actually probably make me do it more, I think.	Cognitive<br><br>Behavioural	Initial Behavioural (micro) → Cognitive → Subsequent Behavioural (micro)
Lack of clarity / guidance	Negative	Capability (psych)<br>Motivation (auto)	Memory..., Knowledge	6	“There's no sort of introduction thing really. So it could've possibly done with an introduction page to start with.”	Cognitive<br><br>Behavioural	Initial Behavioural (micro) → Cognitive → Subsequent Behavioural (micro)

Technical difficulties	Negative	Opportunity (phys)	Envr.	2<br><br>11	And also with that was, if you accidentally click on the wrong date, you weren't able to unclick it. They ask you, you get a notification in the morning telling you to do yesterday's activity<br><br>Yeah, it was fairly easy. I did come across a problem. I don't know. I guess I'd been using it a couple of months and then it just froze on me.	Behavioural	Context → Subsequent Behavioural (micro)
Unable to use	Negative	Capability (phys)<br>Opportunity (phys)	Envr.<br><br>Skills	1<br><br>11	My kids are a bit young to do it for themselves because they're four and seven.<br><br>They tried a few with the little kind of game things that run their [inaudible 00:07:14] or something where you pull down that thing. My eldest son he's 18 fairly understood it, but the younger one really didn't.	Cognitive<br><br>Behavioural	Initial Behavioural (micro) → Cognitive → Subsequent Behavioural (micro)
Perceived impact							
Accountability (to HCP)	Positive	Motivation (auto)<br>Motivation (ref)<br>Opportunity (soc)	Social<br><br>Beliefs consq.	1<br><br>7	And also someone to be accountable for. So, someone to say, why didn't you have fruit on that day then? And because it's hard to explain it sometimes isn't it? If the truth is because I was a bit lazy. And if you know you've got to say that to someone next week, you're more likely to go and have an Apple love.<br><br>If I knew that somebody was there and had gone, "Oh, I see you haven't moved very much, and maybe you should get out and about, it'd be really healthy for the children." I think that'd be the key thing, the benefits for me are great, but I think highlighting the benefits for the children, if they go, "Oh, I see you haven't been out with the children for a bit." I'll be like, "Ah, you're right. Okay, yeah. Yeah, we will do that."	Affective<br><br>Cognitive<br><br>Behavioural	Initial Behavioural (micro) → Cognitive → Affective → Subsequent Behavioural (micro)<br><br>Behavioural (micro) → Cognitve → Behavioural (macro)
Comfort / trust with HCP	Positive	Motivation (auto)<br>Opportunity (soc)	Social<br><br>Emotion<br><br>Beliefs consq.	6<br><br>5	But if you didn't trust each other to start with, then I could see you might be thinking, well they're checking up on me or all this.<br><br>But, because I just found the app really tedious at times, I knew that I had to update it and I just couldn't be bothered, honestly, to go and update it, I don't know, I feel it would make me out to be somebody that's not interested in my children's nutrition, and not worried, not interested in how I'm feeding them and stuff like that. It portrayed me to be somebody that's not taking an interest in their food and wanting to keep the healthcare professional updated. And so, that wouldn't be the truth because I would be doing my part. I just wouldn't be updating it on the app, but they wouldn't know that	Affective<br><br>Behavioural	Context → Affective → Behavioural (micro)

Privacy / security concerns	Negative	Motivation (ref)	Beliefs consq.	13	I would think initially I would probably be quite skeptical about how secure it was, because anything to do with that, I'm really into the privacy. ... You need to know the information behind it and who's made it and where the information is stored and how it's used as well, definitely.	Cognitive<br><br>Behavioural	Initial Behavioural (micro) → Cognitive → Subsequent Behavioural (micro)
Useful feedback (from HCP)	Positive	Capability (psych)<br>Motivation (auto)<br>Motivation (ref)<br>Opportunity (soc)	Reinforce-ment<br><br>Beliefs cap.<br><br>Knowledge<br><br>Social	7<br><br>2	[If] she had a healthcare checkup, and actually, had they then interacted with us on the app saying, "Here's some really good things to do. Oh, I see you haven't been moving very much, do you need some more ideas of what to do?" Particularly during the times that we've had recently, actually, I think it may have been quite useful.<br><br>Whereas if they had access to the information, it would be a lot easier, I feel. They could be, "We need you to be getting on with this. We need you to be doing that. I see you're doing good with this." Also it's like they can see that you're doing stuff and there's no progress or the results they're looking for, but yet you're doing the work but there's no progress, then they can be seeing something's not working here, we have to take a different approach.	Cognitive<br><br>Behavioural	Context → Cognitive → Subsequent Behavioural (micro)<br><br>Context → Cognitive → Behavioural (macro)
Already doing behaviour	Negative	Motivation (ref)	Intentions / goals	4	We live very close to school, so it's easiest to walk. So that thing that I put in as our first goal about walking to school every day. We were going to use it every day, anyway. Then I suppose it's getting a bit more creative about what we could put on as another goal above that because I suppose I could put on his PE lessons, but he was already doing that every day.	Behavioural	Context → Behavioural (macro)
Bad weather	Negative	Opportunity (phys)	Envr.<br><br>Emotion<br><br>Beliefs consq.	12<br><br>15	And, there was definitely more motivation to do it when the weather was a bit warmer, but now it's dropped so cold, there is less.<br><br>So once we've had a family meal it's dark, the children aren't wanting to go back out. I'm not really wanting to go back out. It's cold.	Affective<br><br>Cognitive<br><br>Behavioural	Context → Affective → Behavioural (macro)<br><br>Context → Cognitive → Behavioural (macro)
Covid-19	Negative	Motivation (auto)<br>Opportunity (phys)	Envr.	10<br><br>11<br><br>3	I guess it's been harder because we now don't go to school, so we don't walk to school or anything like that.<br><br>It's not always easy, especially with lockdown when you don't feel great anyway, trying to look at healthiest snacks as well.<br><br>And, just as well with the lockdown, a lot of the stuff you couldn't have done like swimming and that kind of thing.	Affective<br><br>Behavioural	Context → Behavioural (macro)<br><br>Affective → Behavioural (macro)

Family dynamics	Either	Opportunity (phys)<br>Opportunity (soc)	Social<br><br>Skills	13<br><br><br><br>14<br><br><br>15	I think for him, because he was at the age where quite a lot of groups and things we would have joined you had to be six or seven, and some of it is a safety, like riding his bike and things like that, he had more practice. He is not exactly road safe, so it's things like that.<br><br>He would eat loads of fruit, and generally most food, but he was so hard to get to eat vegetables. And he just refused to eat anything.<br><br>We have kiddie teasing in our house, I suppose older brother to younger brother teasing the other one. And the fat word does get used occasionally.	Behavioural	Context → Behavioural (macro)
Lack of accessability / affordability	Negative	Opportunity (phys)	Envr.	1<br><br><br><br>8<br><br><br>10	My goal was to get outside more, be outside every day. We don't have outside space for us. It would be easier to let the kids play in the garden, but we don't have a garden. So, it's an effort to do it every day.<br><br>Because I know from doing the supermarket shop, it's cheaper and easier to get frozen food and unhealthy snacks than it is to get healthier ones.<br><br>Because if some of the suggestions, say we live in xxxxxx, it's not got the widest variety of activities that you can do around here. And therefore some of the ideas were good, but there's just nowhere around here that we'd be able to do them.	Cognitive<br><br>Behavioural	Context → Behavioural (macro)<br><br>Cognitive → Behavioural (macro)
Lack of motivation	Negative	Motivation (auto)<br>Motivation (ref)	Emotion<br><br>Intentions, Beliefs consq.	4<br><br><br><br><br>6<br><br><br>11	Because we all know if we're tired, you're more likely to want to eat more because you feel you need something to give you a boost. It's like, "Okay, well I'm going to go and have something like a bar of chocolate because it's going to make me feel a bit better.<br><br>You know when you don't really want to do something yourself, you can't really find that enthusiasm to assist your child with it as well.<br><br>Just because it can be so easy just to grab something quick rather than, okay, I'm going to prepare a piece of fruit for my children and they will eat the fruits.	Affective<br><br>Cognitive<br><br>Behavioural	Context → Behavioural (macro)<br><br>Affective → Behavioural (macro)<br><br>Cognitive → Behavioural (macro)
Lack of skills / ability	Negative	Capability (psych)<br>Capability (phys)	Skills	8	But I think they eating side of it is harder because it's just easier to buy your junk food, it's cheaper and it's easy to put... I mean, I do it myself sometimes, to put something in the oven to cook rather than cook from scratch. And some people might not necessarily have the skills.	Cognitive<br><br>Behavioural	Context → Behavioural (macro)<br><br>Cognitive → Behavioural (macro)

Lack of time	Negative	Opportunity (phys)	Envr.	5<br><br>15	Like, I'm a parent and finding the time to cook a healthy, nutritious meal, it's limited at times.<br><br>We've literally been stuck at home with the children. I work in the afternoon, so once we've done schoolwork I find our time is very limited. I go out to work and I'm not back till half five/six	Behavioural	Context → Behavioural (macro)
Awareness of behaviour	Positive	Motivation (auto)<br>Motivation (ref)<br>Capability (psych)	Goals, Beliefs cap.<br><br>Memory..., Behav reg.	2	We've got habits and this kind of brings it forward for you to actually acknowledge what you're doing and what you're not doing, and to help you to correct it.	Cognitive<br><br>Behavioural	Behavioural (micro) → Cognitive → Behavioural (macro)
Doing as a family	Positive	Motivation (auto)<br>Opportunity (soc)	Social<br><br>Emotion	3	It just felt like something that we were all doing together. Soon as you downloaded it, it did feel sort of like a whole family thing that you were going to work, and do challenges together, and that's what I liked about it.	Affective<br><br>Behavioural	Behavioural (micro) → Affective → Behavioural (macro)
App suggestions (for behaviours)	Positive	Capability (psych)	Knowledge	8<br><br>1	I thought the useful links were good that led you to other activities and different websites or places that you could go and visit because I think if you're not a family that's necessarily an outdoorsy family, you might not even know where to start with, where to go.<br><br>But actually, stuff like choose a bit of fruit instead of a biscuit. It's such a sensible and obvious thing. And I couldn't see the wood for the trees for a bit if you know what I mean? And it was helpful in that way.	Cognitive<br><br>Behavioural	Behavioural (micro) → Cognitive → Behavioural (macro)
Goal setting support	Positive	Capability (psych)	Goals<br><br>Knowledge<br><br>Beliefs cap.	1<br><br>8	I liked that they were simple, and I liked the way that they were suggested. Otherwise, I would have been sitting there wondering, I don't know what a goal should be for this.<br><br>I think the barrier thing was quite good as well because it makes you realize that what you think is a barrier, isn't really a barrier. So my barrier is we're always late anyway, because I'm always on the last minute so it's just quicker to jump in the car. Well, in fact, that's just really not a very good excuse at all really, just get up 5, 10 minutes earlier.	Cognitive<br><br>Behavioural	Behavioural (micro) → Cognitive → Behavioural (macro)

Notifications	Either	Capability (psych)<br>Motivation (auto)<br>Motivation (ref)	Memory...<br><br>Emotion, Reinforce-ment	12<br><br><br><br><br><br><br>7	I think the reminders would have definitely been helpful. So, if there'd been a prompt to say, have you hadn't done any exercise today, get moving or do 10 star jumps, a bit like, obviously it's not the same, but if you've not moved for a while, your Fitbit it'll buzz and say, "do 10 star jumps", maybe if its a reminder, just pop up saying, "Have you had any exercise today" or "Challenge: Do 10 star jumps" or something like that?<br><br>I think so, because there were some times when actually we weren't going to do anything, and then seeing the reminder come up made me go, "Oh, yeah, I'm recording that. Oh, okay, maybe..."	Cognitive<br><br>Behavioural	Behavioural (micro) → Cognitive → Behavioural (macro)
Useful feedback	Positive	Capability (psych)<br>Motivation (auto)<br>Motivation (ref)	Reinforce-ment<br><br>Behav reg., Knowledge	9<br><br><br><br><br><br><br>13	I think one day I told it, like I said, I wanted to drink two liters of water. And then I think I said a liter and it was like, "You're not quite there." I was like, "Oh, I'm not?" Oh yeah, I'm not, because I told you that I wanted two liters. So it was good because then it triggers something in your brain to think, "Why is he not telling me well done?" Well, did I set that? That also made me really look at what my goals were.<br><br>So for the riding our bikes, we've gone weeks without doing it, but it might be good for something to say, "Hey, we've noticed you've not done that. What was your barrier? How can you overcome that? What would help you?"	Cognitive<br><br>Behavioural	Behavioural (micro) → Cognitive → Behavioural (macro)
