# Supplementary material for: Mapping the Process of Engagement With Digital Health Interventions: A Cross-Case Synthesis
Source: Mayo Clin Proc Innov Qual Outcomes. 2025 May 27;9(3):100625. doi: 10.1016/j.mayocpiqo.2025.100625 (PMC12158608; doi:10.1016/j.mayocpiqo.2025.100625)
Supplement: Supplemental Table 4 [file mmc9.pdf]

Supplemental Table 4. Case Study 2: Thematic framework mapping factors to engagement components and patterns of engagement

Organising theme	Theme	Ppt	Quotes	Code	Engagement component	Pattern of engagement
Factors associated with engagement	Tool was exciting and a relief because of accessible support and expected benefit	2<br><br>1<br><br>4	So I was just really curious on what kind of things they would be available to use.<br><br>So the whole idea that it's an app and not another person is that... I'm not bothering anyone... why I don't talk about things is because I don't wanna burden people with my issues.<br><br>So I feel like it could be useful for many different people who are waiting for help.	Anticipated affective	Affective	Affective → Initial Behavioural (micro)<br><br>Cognitive → Initial Behavioural (micro)<br><br>Cognitive → Affective → Initial Behavioural (micro)
				Curious about trial / new tool	Affective	
				Excited about using	Affective	
				Worried about lack of support on wait list	Affective	
				Relief	Affective	
				Liked accessibility	Affective	
				Accessing help without bothering anyone	Affective	
				No reluctance	Affective	
				Perceived effectiveness - anticipated	Cognitive	
				Expected emotional benefits / guidance	Cognitive	
				High expectations of chatbot	Cognitive	
				No particular expectations of ability - not expecting the world	Cognitive	
	Tone affected user connection with app	4<br><br>3	I like that it was very judgment free, like it didn't like point fingers.<br><br>It would ask you why you didn't check in in the morning. There was like 3 responses that it gave you and if you said any of them, it would come back	Tone	Affective	Initial Behavioural (micro) → Affective → Subsequent Behavioural (micro)
				Positive, genuine, non-judgmental	Affective	
				Tone could be misinterpreted as condescending	Affective	
				Liked mascot (approachable)	Affective	

			with like quite a condescending perhaps response, but this could just be a unique issue because I have like difficulties understanding tone.	Not convinced about mascot	Affective	
	Limited chatbot frustrated users	1<br><br><br><br><br><br>2	I know it's not the same as having a person there... but I found it becoming frustrating because like I say, I could predict it and if I said no to something it would ask me again or and... there were inconsistencies as well.<br><br>It does affect me in terms of how much I access the app because unless I really want to use it, I probably just ignored it.	Frustration with chatbot	Affective	Initial Behavioural (micro) → Cognitive → Subsequent Behavioural (micro)<br><br>Initial Behavioural (micro) → Cognitive → Affective → Subsequent Behavioural (micro)
				Lack of personal connection	Affective	
				Tedious	Affective	
				Made inputs simple for chatbot	Cognitive	
				Perceived effectiveness - experienced	Cognitive	
				Different from discussing with a person	Cognitive	
				Confidence decreased with chatbot	Cognitive	
				Inconsistencies from app / chatbot doesn't listen	Cognitive	
				Limited / predictable / repetitive algorithm	Cognitive	
				Micro engagement	Behavioural	
				Issues/frustrations with chatbot affected use	Behavioural	
	Trustworthiness shaped by source credibility and personal experience	1<br><br><br><br><br><br><br>4	I would have had deep confidentiality concerns because and I'm putting all my trust in the NHS there and hoping they've done due diligence, but you know they're a government entity, so they'd be foolish not to.<br><br>*Did you find the program and the resources credible and trustworthy?* Yes, the previous exercises were really helpful actually, in moments and stress.	Privacy concerns associated with what's shared with app	Affective	Initial Behavioural (micro) → Affective → Subsequent Behavioural (micro)<br><br>Initial Behavioural (micro) → Cognitive → Affective → Subsequent Behavioural (micro)
				No concerns	Affective	
				Trustworthy	Affective, Cognitive	
				Trusted because NHS / sources cited	Affective, Cognitive	
				Experience of it helping improves credibility	Cognitive	

	Easy to use but a bit overwhelming	2	I think the way it was presented for us to follow through app is quite easy and just really straightforward.So there was no learning curve and trying to do the exercises.	A bit overwhelming	Cognitive	Initial Behavioural (micro) → Cognitive → Subsequent Behavioural (micro)
				Easy to use	Cognitive	
				Confident in ability to use	Cognitive	
		4	Not like really difficult, but like, and the overwhelming is the layout I think. Because it's not sectioned off, [the content is] kind of just there.	No costs	Cognitive	
				Short videos with timings were good	Cognitive	
				Categorisation might hide useful content	Cognitive	
	Reminders helped prompt use but app was easy to ignore	2	I don't think anything cuts my attention as much.	Micro engagement	Behavioural	Initial Behavioural (micro) → Cognitive → Subsequent Behavioural (micro)
		3	To be honest, I just kind of it was just kind of one of the things where it was very easy to ignore, which I think as I was saying earlier about people like there's the option to just not open the app at all.	*Easy to ignore / nothing grabs attention*	Behavioural, Cognitive	
				Reminder prompted use	Behavioural, Cognitive	Initial Behavioural (micro) → Affective → Subsequent Behavioural (micro)
		3	If you're in the heat of anger, you don't really have the time to do that, or you just can't be bothered to do that.	*Tailoring reminder times would be good*	Behavioural, Cognitive	Context → Affective → Behavioural (micro)
				No incentive (esp. if emotional)	Affective	
	Suggestions for improvement	3	I think like even having just a rotating list that changes every day of just why don't you try this and then at the end saying is that worked or if it didn't work you got like delivery like saying like ohh did that work or did it not work because the only really thing it asks you is right your experience I don't think anybody is going to do that because it's just gonna be reading the app and not the exercise itself.	App suggestions helpful - keep fresh	Cognitive	Initial Behavioural (micro) → Cognitive → Subsequent Behavioural (micro)
				Create a personal library	Cognitive	
				Video not always convenient - other suggestions	Cognitive	
				Macro engagement	Behavioural	Behavioural (micro) → Cognitive → Behavioural (macro)
				Want more training for using skills outside app	Behavioural, Cognitive	
		4		Perceived effectiveness - experienced	Cognitive	

Perceptions of potential usefulness in clinical pathways	Range of resources were useful for users	1	The chat function was really good because it would help you. It wouldn't just reflect on it. It would help you like reset your mindset and most like it would make you rethink how you feel or something.<br><br>Taking a look at the demo dealing with whatever depression, anxiety with whatever mental health issues it's, it's certainly it a really good introduction to that and lots of good different resources and covering different topics to to give people the confidence without feeling they're being railroaded into something to to actually go out and and do a little bit more investigation themselves.	*Confident in ability to support*	Cognitive	Initial Behavioural (micro) → Cognitive → Subsequent Behavioural (micro)<br><br>Initial Behavioural (micro) → Cognitive → Affective → Subsequent Behavioural (micro)<br><br>Behavioural (micro) → Cognitive → Behavioural (macro)
				Having something to talk to was good	Cognitive	
				Good range of activities	Cognitive	
				Liked breathing aspect	Cognitive	
				Resources were helpful (at least short-term/for some)	Cognitive	
				Sleep benefits	Cognitive	
				Chats helped	Cognitive	
				Chatbot helped reflection	Cognitive	
				Helped prompt previously learned techniques	Behavioural, Cognitive	
				Liked videos	Affective	
	App did not meet all needs; not long-term solution or replacement for therapy	2<br><br>1	Uh, I think I would give it on a scale of 10, maybe it's six cause for me definitely the exercises help, but if I'm looking for uh uh, not journaling, but like that conversation to help me ease mood or something.When you're like having like an episode or something, it definitely not gonna help uh with the current limitations and how the chat works.<br><br>But for me, I needed something more. I needed 'Why is this happening?' kind of thing ... rather than a quick sometimes this happens, sometimes that happens and I'm thinking well, no, that's not the case here.	Perceived effectiveness - experienced	Cognitive	Context → Behavioural (macro)<br><br>Initial Behavioural (micro) → Cognitive → Subsequent Behavioural (micro)
				Benefits depend on user not just tool (e.g. motivation, experience with therapy, mental health issues)	Cognitive	
				Not effective	Cognitive	
				Not for long-term / replacing therapy	Cognitive	
				Premium better than free?	Cognitive	
				Got some of what they wanted out of the app	Cognitive	
				Want to understand issues	Cognitive	
		3		Micro engagement	Behavioural	

How patients used the app	Varying patterns of use for individuals	2<br><br>4	And so I said that I'm frustrated and it gave me an 8 minute video to watch. And in my head, I was like, well, I didn't have time to do that.<br><br>Because I feel like if I have, I'm like forced to always check in like in the morning and it becomes like too tedious and I will not use it like genuinely. I would just like check in for sake of checking in, so I'm not really using it that it's beneficial for me and it's more like I'm being forced to do it and out of my own.<br><br>I did like the check-ins that it does. So it's like what will happen in the day or what might happen in the day and at the end of the day what has happened that day.	*Don't always have time*	Behavioural, Cognitive	Context → Subsequent Behavioural (micro)<br><br>Context → Behavioural (macro)
				Not using regularly	Behavioural	
				Used in quiet moments of day	Behavioural	
				Used to satisfy immediate need	Behavioural	
				Built into daily routine	Behavioural	
				Lifestyle and other factors could affect use	Behavioural	
				Didn't use all features	Behavioural	
	Decrease in use over time	3	I think I started to use it less. You you get the information you need and then you leave.	Micro engagement	Behavioural	
				Decrease in use over time	Behavioural	
	Minimal transfer of skills beyond app	1	But yeah, it's more, you know, I see something. Satisfies that need and then it's forgotten. I would have liked if I was able to to retain more of it rather than kind of being reliant on it.	Macro engagement	Behavioural	
				Not much outside app	Behavioural	
				Once you have what you need, don't need app	Behavioural	
				Used skills beyond app	Behavioural	
