# Supplementary material for: Mapping the Process of Engagement With Digital Health Interventions: A Cross-Case Synthesis
Source: Mayo Clin Proc Innov Qual Outcomes. 2025 May 27;9(3):100625. doi: 10.1016/j.mayocpiqo.2025.100625 (PMC12158608; doi:10.1016/j.mayocpiqo.2025.100625)
Supplement: Supplemental Table 5 [file mmc10.pdf]

Supplemental Table 5. Case Study 3: Thematic framework mapping factors to engagement components and patterns of engagement

Theme	TFA constructs	Codes	Ppt	Quotes	Eng. component	Pattern of engagement
Expectations prior to the call	Affective attitude (anticipated)	Negative<br>No expectations or unsure<br>Positive or no concerns	3	Well, I was worried in case it was a bit like press one, press two, all this kind of relationship when actually there is nothing... It could be frustrating. I was worried in case it could be frustrating, but it wasn't.	Affective<br>Cognitive	Cognitive → Initial Behavioural (micro)<br><br>Cognitive → Affective → Initial Behavioural (micro)<br><br>Context → Initial Behavioural (micro)
	Burden	Anticipated - could be frustrating	16	Well, I thought the rationale for it was very good really, because it's quite a interventionist procedure, a kind of cataract removal. So to have a source of advice, guidance, information, was a good thing. So I was expecting to give feedback and get information and felt positive.		
	Perceived effectiveness (anticipated)	Confidence<br>No expectations<br>Uncertain about Dora R1's ability	7	So I'm coming from a position of a lot of confidence with anything that comes out of the NHS really. I'm taking the position that anybody from the NHS contacting me with a thing like this is starting from a high bar. So I would have no reason to be suspicious of it really.	Cognitive<br>Behavioural	
			1	Well, I think I have to be honest and say I didn't really know what to expect... I wondered if Dora R1 would understand my answers.		
Ease of use	Burden	Easy to use	14	It didn't take a great deal of effort. It was quite straightforward.	Cognitive	Initial Behavioural (micro) → Cognitive → Subsequent Behavioural (micro)
			15	So the liked bit is it's very quick and efficient.		

	Intervention coherence	Understand call procedure<br>Understand tech (at least broadly)<br>Didn't have clear understanding	1<br><br><br><br><br>13	So I think what I realized that Dora R1 would've been programmed by a clinician to ask certain questions, but if there was a query about something, then Dora R1 would not have had the wherewithal to further probe what that problem might be.<br><br>I don't know how you interpret the information or how the information is used there.	-	
Usability issues or concerns	Affective attitude (experienced)	Uncertainty on how to respond<br>What happens if call missed<br>Not much time to think	13<br><br><br><br><br>9	I think it is quite precise, but like I say, I couldn't quite know like from what perspective is she answer the question for me. *So there was a bit of uncertainty than it sounds like about how best to respond, is that right?* Yeah, correct.<br><br>If you were told you only had two seconds to answer everything or two seconds to write something down, it's all instant, instant, instant, you wouldn't, I feel get the right picture, but that's the sort of thing that was happening with Dora R1, you were just... doing it, doing it, doing it, answering.	Affective<br>Cognitive<br>Behavioural	Initial Behavioural (micro) → Cognitive → Affective<br><br>Initial Behavioural (micro) → Cognitive → Subsequent Behavioural (micro)
	Burden	A bit too long<br>Give Dora R1 simple answers	5<br><br><br>3	Well, obviously just perhaps a bit slower, and simple words. Like talking to a toddler really, isn't it?<br><br>Knowing it was a machine, you keep it simple and you go to the essential, which is actually a good thing.	Cognitive<br>Behavioural	
	Self-efficacy	Low - difficult	13	Quite difficult. You can't because I don't know if I'm talking to a person or a machine. A person, a machine, what is it, a computer?	Cognitive	
Accessibility	Perceived effectiveness (experienced / long-term)	Didn't feel understood - e.g. Language or accent barrier<br>Difficult or inappropriate for certain types of patients - accessibility	18	She was very fluent in what she has to say, even though she had an accent. Because she had an accent, I was listening more deeply, just to get the t's and the I's. So yeah. Communication was okay.	Affective<br>Cognitive	Initial Behavioural (micro) → Cognitive → Subsequent Behavioural (micro)

	Burden	Hearing and Dora R1's accent is a potential difficulty	11	I was extremely distressed by the whole experience. Now that may be because I'm autistic, and so I process things neurologically very differently from a neurotypical person. So if this Dora R1 thing is asking questions, which from an AI point of view, it would be asking closed questions. They will only get, if they're on to an autistic person, they will only get a certain answer... and I think that's dangerous.		Initial Behavioural (micro) → Cognitive → Affective → Subsequent Behavioural (micro)<br><br>Context → Behavioural (micro)<br><br>Context → Cognitive → Behavioural (micro)
Positive attitudes towards and confidence in Dora R1	Affective attitude (experienced)	No concerns<br>Good experience<br>Neutral experience (fine) - no particular likes or dislikes<br>Reassuring to have 'safety net'	20<br><br>8<br><br>6	Well, I probably would say no follow up, really. Oh, I don't know. Maybe Dora R1, just because that is reassuring, or would be reassuring, to some people I think.<br><br>Well, the questions she asked, I mean, I can't remember offhand, but it made me more comfortable after the operation.<br><br>I didn't really [have any concerns], because there's nothing to lose and a lot to gain if it actually works.	Affective<br>Cognitive	Initial Behavioural (micro) → Affective → Subsequent Behavioural (micro)<br><br>Behavioural (micro) → Affective<br><br>Behavioural (micro) → Cognitive → Affective
	Self-efficacy	High - confident, comfortable expressing self	8	Well, it was quite easy. I felt comfortable with it, with her.		
	Perceived effectiveness (experienced)	Perceived effective, satisfactory (confidence in Dora R1)<br>Potential for harm - not likely	10<br><br>17<br><br>19	She answered the questions that I asked to my satisfaction... It was good. It was clever.<br><br>I think it would work very well. It was clear. The question that he asked was clear and direct.<br><br>Harm? I don’t think so... can’t see any reason why it would harm anybody	Cognitive	

Importance of human aspect	Perceived effectiveness (experienced)	Dependent on complications, concerns	1<br><br>11<br><br>2	I don't know how good it would be if you had a very nervous patient or a very worried patient, or somebody who had not expected the outcome that had actually happened post surgery.<br><br>I cannot for the life of me, see how a robot can possibly be of any use in talking, well, I use the term loosely, in talking to patients. The situation is far too complex for artificial intelligence, and I use the word intelligence very loosely as well.<br><br>But my own view is that in probably 90% of cases, the end result is perfectly satisfactory for the people who've used it, but there may be 10% where you're not necessarily getting from it the full picture.	Affective	Initial Behavioural (micro) → Affective → Subsequent Behavioural (micro)<br><br>Behavioural (micro) → Cognitive → Affective<br><br>Behavioural (micro) → Affective<br><br>Initial Behavioural (micro) → Cognitive → Subsequent Behavioural (micro)<br><br>Context → Cognitive → Affective → Behavioural (micro)
	Self-efficacy	Depends	15	Partially. So as it happened, everything was fine. So I felt able to tell it that. But if it hadn't been or if there was... If I'd had a worry, I don't think... I'm not sure I would've been reassured.	Affective	
	Opportunity costs	Lack of human element (empathy, reassurance, etc)	10<br><br>9<br><br>11	If Dora R1 had rung me up, and I'd had a cataract operation, and I had lost the sight in my right eye, how would Dora R1 have coped with that? You could talk about the facts, but have they got enough emotion into the artificial intelligence to be able to be sensitive to people's feelings.<br><br>I thought it was fine, but it felt abstracted. It wasn't personal. It really came through that way.<br><br>*What do you think you lost or gave up by having a call with Dora R1 instead of an in-person follow-up assessment?* Humanity.	Affective	
	Perceived effectiveness (experienced)	Dora R1 as first step - clinician as backup is important	3	Rather than wasting time to have an appointment to go to a hospital just to be told that you don't have any problems. One, you already know that you don't have any problems. I think that can also be useful, provided that you know that in case you have problems or in case you want to talk to a human, being seen that's still available.	Cognitive	

			16	And for Dora R1 to develop to that degree of sophistication might not be cost productive, but I mentioned [my complication] because I think it's quite a good example of why we still need readily available clinician backup.		
	Affective attitude (experienced)	Perfunctory, brisk<br>Impersonal, remote feeling<br>Bad experience	10<br><br>7	I always feel a bit remote and lonely when I'm talking to a machine but yeah, I mean, I think that this is the way things are going.<br><br>Well, almost like talking to a person, but not quite. It didn't quite have the warmth of a conversation with a person.	Affective<br>Behavioural	
	Ethicality	Can't treat people as objects - need human aspect	11	Yes, I do [think it could harm]. I was extremely distressed by the whole experience... I think when you are dealing with health and patients, I don't think you can put them through a tick box exercise because it requires a level of being human, I suppose.		
Concerns about Dora R1's effectiveness	Affective attitude (experienced)	Cannot have two-way communication to discuss, question, clarify	14<br><br>2	Well, you're more likely to be able to develop a two-way exchange if you are speaking to a person.<br><br>I'll give you one minor example. When I had the first eye done... I could see a slightly brownish area in my vision. My thoughts were, I don't think this is anything to worry about... but face to face, I might have raised that. Whereas not all the questions really seemed to encourage me to include that in it.	Cognitive<br>Behavioural	Initial Behavioural (micro) → Cognitive → Subsequent Behavioural (micro)<br><br>Initial Behavioural (micro) → Cognitive → Affective → Subsequent Behavioural (micro)<br><br>Initial Behavioural (micro) → Affective → Subsequent Behavioural (micro)
	Affective attitude (experienced)	Covers current time point only<br>Not taken seriously	1<br><br>15	I personally had no concern about talking to Dora R1, but I could understand that if Dora R1 was going to replace a face-to-face interview with the consultant or one of his team, then I might feel a bit concerned in case my worries or my fears weren't taken seriously.<br><br>Or something that isn't happening now, but did happen and you're worried it might happen again so that... So there isn't a yes or no answer, because it's like, again, back to the redness, "Well, no, [my eyes are] not red, but they were and I'm a bit	Affective<br>Cognitive<br>Behavioural	

				worried about it. And do you think... Dora R1, is that going to be all right, Dora R1?" You know?		
	Burden	Had to interpret questions	4	And if I didn't understand it, and I interpreted what I thought the question might have meant.	Cognitive	
	Perceived effectiveness (experienced / long-term)	Perceived NOT effective (no confidence in Dora R1)<br>Potential for harm - possible<br>Not good for long-term application	2<br><br>4<br><br>11	When you've got a person in front of you, you can express a question in a particular way and the person can analyze that and give you an answer. AI relies on the programming of the AI responses. It's not as flexible as a human is.<br><br>I didn't think that I had all that much confidence. I wanted to ask the questioner who was a machine, not a person, what do you mean by that? I couldn't do that.<br><br>Personally, on a scale of one to 10, with 10 being excellent and zero being completely useless, I'd have to put it at zero. I think if you've been through a surgical procedure, you do NOT want to be contacted by a robot.	Affective<br>Cognitive<br>Behavioural	
	Perceived effectiveness (experienced)	Concern that non-verbal info or nuances missed<br>Hard to catch Dora R1's failures	1<br><br>16	Because when you speak face-to-face, or when you're talking to somebody, fear or worry or whatever, might either be visible on your face or in your voice. And that obviously didn't apply to Dora R1.<br><br>My main criticism, and I suppose it's inevitable in an automated system, it doesn't provide some nuances and shades of opinion. I was slightly nonplussed, upset, when it said about this irritation or, like I've told you, just to the eyes, yes or no. And I said I'd had it intermittently.	Affective<br>Cognitive	
Potential benefits more broadly	Perceived effectiveness (long-term)	Could have benefits if used more extensively (cost saving, data analysis, minimise human shortcomings, saves time, etc.)	2	I'm delighted that they're doing a proper follow-up...There are all sorts of operations that happen in hospitals and I wonder if eye surgery's the only one where Dora R1 is useful as a followup. It probably could be useful in other situations.	-	-

Willingness to use Dora R1	Affective attitude (experienced)	Preference Dora R1 or F2F	16<br><br>17<br><br>19	Well, if I felt things were going okay, and I didn't want the hassle of being in the hospital or going down there, I settle for Dora R1.<br><br>I feel more comfortable talking to [Wysa] than a person telling the truth. When you're talking to a person, sometimes you have to answer the question over again. [Wysa'a] straight, clear and direct.<br><br>I think face to face would be better for me, I get lost in between, sometimes I don't understand the questions, if I'm face to face I'm able to ask to explain it to me better.	Affective<br>Cognitive<br>Behavioural	Initial Behavioural (micro) → Cognitive → Subsequent Behavioural (micro)<br><br>Context → Behavioural (micro)<br><br>Initial Behavioural (micro) → Affective → Subsequent Behavioural (micro)
	Affective attitude (experienced)	Willing to use again	4<br><br>6<br><br>13	I would tolerate it, if that's the only way I could make a contribution after the operation.<br><br>Well, I've got another [operation] this month. So if she wants to ring me up after having the op, fine.<br><br>Happy to use Dora R1 again, yeah.	Affective<br>Behavioural	
	Opportunity costs	Lost time<br>Nothing gained<br>Nothing lost except short phone time	1	I didn't really lose anything except for 20 minutes or so of a telephone call.	Cognitive<br>Behavioural	
Suggestions	Other	Ability to add more info or detail<br>Ability to schedule and reschedule<br>Accessible to all<br>Don't use it<br>Monitor and update (keep it learning)<br>More clarity - objective, questions<br>Paper version<br>Questions in advance<br>Reminders	3<br><br>16<br><br>2	So to make sure that if you miss the call, how to alert the system that you are available.<br><br>See if the advice responses, follow-up questions could be developed to make it even more comprehensive.<br><br>To be honest with you, I wonder if a paper version of Dora R1 would be perhaps slightly more effective because people would have more time to actually think about the answers they want to give.	Affective<br>Cognitive<br>Behavioural	Context → Behavioural (micro)<br><br>Context → Cognitive → Affective → Behavioural (micro)

		System set up to include Dora R1 with safety net of human				
