# Supplementary material for: Mapping the Process of Engagement With Digital Health Interventions: A Cross-Case Synthesis
Source: Mayo Clin Proc Innov Qual Outcomes. 2025 May 27;9(3):100625. doi: 10.1016/j.mayocpiqo.2025.100625 (PMC12158608; doi:10.1016/j.mayocpiqo.2025.100625)
Supplement: Supplemental Table 6 [file mmc11.pdf]

Supplemental Table 6. Pattern-matching of theoretical and empirical mappings of engagement components

**Patterns of engagement**	***Hypothesised***	**Case 1: NoObesity**	**Case 2: Wysa**	**Case 3: Dora R1**
Evidence for components affecting initial micro behavioural engagement				
Affective → Initial Behavioural (micro)	*X*		X	
Cognitive → Initial Behavioural (micro)			X	X
Affective → Cognitive → Initial Behavioural (micro)	*X*			
Cognitive → Affective → Initial Behavioural (micro)			X	X
Context → Initial Behavioural (micro)				X
Evidence for components affecting subsequent micro behavioural engagement				
Initial Behavioural (micro) → Affective → Subsequent Behavioural (micro)	*X*	X	X	X[a]
Initial Behavioural (micro) → Cognitive → Subsequent Behavioural (micro)	*X*	X	X	X
Initial Behavioural (micro) → Affective → Cognitive → Subsequent Behavioural (micro)	*X*			
Initial Behavioural (micro) → Cognitive → Affective → Subsequent Behavioural (micro)		X	X	X[a]
Context → Subsequent Behavioural (micro)		X	X	X[a]
Context → Affective → Subsequent Behavioural (micro)		X	X	
Context → Cognitive → Subsequent Behavioural (micro)	*X*[b]	X		X[a]
Context → Affective → Cognitive → Subsequent Behavioural (micro)	*X*[b]			
Context → Cognitive → Affective → Subsequent Behavioural (micro)				X[a]
Evidence for components affecting translation from micro to macro behavioural engagement				
Behavioural (micro) → Affective → Behavioural (macro)		X		
Behavioural (micro) → Cognitive → Behavioural (macro)		X	X	
Behavioural (micro) → Affective → Cognitive → Behavioural (macro)				

Behavioural (micro) → Cognitive → Affective → Behavioural (macro)				
Evidence for components affecting macro behavioural engagement				
Affective (macro) → Behavioural (macro)	*X*	X		
Cognitive (macro) → Behavioural (macro)		X		
Affective (macro) → Cognitive (macro) → Behavioural (macro)	*X*			
Cognitive (macro) → Affective (macro) → Behavioural (macro)				
Context → Behavioural (macro)	*X*[b]	X	X	
Context → Affective (macro) → Behavioural (macro)	*X*[b]	X		
Context → Cognitive (macro)→ Behavioural (macro)	*X*[b]	X		

[a]*As the Dora R1 call comprised one telephone call, 'subsequent behavioural (micro)' in this table can refer both to how initial engagement or context changes subsequent behavioural engagement during the same call and to how engagement with the first call might hypothetically influence engagement with another call, if offered (Figure 2c, Supplemental Table 5)*

[b]*The hypothesised relationships between context and engagement were primarily drawn from the AIM-ACT framework; as this did not break down engagement into the same stages included here, the hypothesised patterns have been selected based on interpretation of the description of 'context' as a component of the framework in the paper*[24]

Note: Blank cells do not indicate that that particular pattern of engagement cannot occur, just that there was insufficient evidence to support it from these three particular case studies; grey cells indicated that this data was not captured in the study.
