# Supplementary material for: Mapping the Process of Engagement With Digital Health Interventions: A Cross-Case Synthesis
Source: Mayo Clin Proc Innov Qual Outcomes. 2025 May 27;9(3):100625. doi: 10.1016/j.mayocpiqo.2025.100625 (PMC12158608; doi:10.1016/j.mayocpiqo.2025.100625)
Supplement: Supplemental Table 7 [file mmc12.pdf]

Supplemental Table 7. List of features and considerations for incorporating them

Feature	Considerations for incorporating into intervention design	Evidence
Context		
Accessibility	The needs of users with different characteristics should be considered during the development of any intervention as this can influence all three components of engagement with both the intervention and the target health behaviour. Where possible, strategies for improving the accessibility of these interventions should be developed in collaboration with people with lived experience as accessibility barriers are likely to be related to the specific context of the target population and the intervention characteristics.	Cases 1-3
Behaviour Change Techniques		
Goal setting and self-monitoring of behaviour	Goal setting and self-monitoring of behaviour can support motivation to engage with the intervention (cognitive and affective engagement) and target behaviour by increasing awareness of a user's current behaviour and highlighting discrepancies with their desired behaviour. Designs incorporating goal setting should enable personalisation of goals to align with users' abilities to avoid triggering negative emotions and disengagement if the goal is too difficult[77].	Systematic review[31], Case 1
Feedback	Delivering encouragement to users can support positive affective engagement and providing advice on adapting goals or strategies to achieve them can support users' cognitive engagement with an intervention.	Systematic review[31], Case 1
Social support	Social support, depending on how it is delivered in an intervention, has the potential to support affective and cognitive engagement by providing a source of motivation to engage with the intervention. For example, social interaction with other users could make an intervention more enjoyable to use. However, social comparison, which might occur as a result of creating a digital community within an intervention, could trigger affective disengagement if users feel demotivated by the comparison or judged by others.	Systematic review[31], Case 1

Rewards	Previous research has highlighted the association of rewards with fun and motivation; however, to support affective and cognitive engagement, the rewards need to be meaningful to the user. One means of achieving this could be to enable users to choose their own rewards and track their progress towards them or to have a digital rewards system that unlocks new content or features (e.g. for children, new avatars or personalisable features).	Systematic review[31], Case 1
Prompts/cues (reminders)	Reminders are a key means of supporting cognitive engagement by drawing users' attention to the intervention. However, badly-timed or overly frequent reminders can cause frustration and irritation, which can lead to affective disengagement. To avoid this, users should be able to personalise reminders to the amount and time that best suits them.	Systematic review[31], Cases 1-3
Design features		
Aesthetic design	Intervention designs should be visually appealing to support affective engagement - although preferences will vary, this broadly includes simple, cheerful styles and colours that are enticing but not overwhelming to look at.	Cases 1 and 2
Tone	The intervention's tone is an important factor in users' connections with it; it should be positive and non-judgmental. In conversational agents, a brisk, impersonal, and mechanical voice or tone could lead to affective disengagement. While clarity is important, developers should also be careful not to be overly simplistic or appear condescending. Tone can be conveyed by the written or verbal content of the intervention and by visual elements (e.g. colour scheme, avatars, etc.).	Cases 1-3
Personalisation	Personalisation is a key element that influences many of the other factors and can help support users' sense of connection with an intervention. Personalisation should be easy to set up and be as flexible as possible, but optional, so that there is not an excessive burden on users who are just wanting to try out the intervention or start using it quickly.	Cases 1 and 2
Usability and guidance	A simple, easy to use intervention that has (or does not require) clear guidance can help support cognitive engagement. A time consuming or overwhelming	Systematic review[31], Cases 1-3

	intervention that requires a high degree of attention and cognitive resources to use and navigate may lead to cognitive disengagement.	
Credibility	Trust and confidence in the intervention can be supported through the use of credible sources (in terms of the intervention content and source).	Cases 2 and 3
Privacy and data security information	Potential concerns around data security and privacy can hinder behavioural engagement; these could be alleviated by providing easily comprehensible information about policies to the credibility of the intervention.	Cases 1 and 2
Gamification	Gamification can support affective and cognitive engagement by providing a fun, enjoyable, and rewarding experience. However, it has similar potential issues as goal setting and social support where excessive difficulty or comparison could decrease motivation and lead to disengagement.	Systematic review[31], Case 1
Other content-related factors		
Interest and novelty of content	Although the intervention should be easy to use and navigate, it should include a large amount and variety of content to sustain users' interest and support cognitive and behavioural engagement. A basic intervention without new content to consume can cause boredom and potentially lead to disengagement.	Cases 1 and 2
Suggestions	Suggestions (e.g. for goals or target behaviours) can support affective and cognitive engagement and macro behavioural engagement by reducing the cognitive effort required to engage or by helping frame the target behaviour as enjoyable.	Case 1
Conversational agent capability	For interventions that include a CA, its capability (as well as its tone) is a key factor that can influence affective, cognitive, and behavioural engagement. Repetition, inconsistencies, inaccuracies, and shallow responses can cause frustration and decrease users' confidence in the ability of the intervention, which can lead to disengagement.	Cases 2 and 3
